# The suppression curve as a new representation of the premature EEG maturation

**DOI:** 10.1186/1471-2202-16-S1-P216

**Published:** 2015-12-18

**Authors:** Ninah Koolen, Anneleen Dereymaeker, Katrien Jansen, Jan Vervisch, Vladimir Matic, Maarten De Vos, Gunnar Naulaers, Sabine Van Huffel

**Affiliations:** 1 Division STADIUS, Department of Electrical Engineering (ESAT), University of Leuven, Leuven, Belgium; 2 iMinds-KU Leuven Medical IT Department, Leuven, Belgium; 3 Department of Development and Regeneration, University of Leuven, Leuven, Belgium; 4 Department of Psychology, University of Oldenburg, Oldenburg, Germany; 5 Institute of Biomedical Engineering, Department of Engineering Science, University of Oxford, Oxford, UK

## 

**Figure 1:**
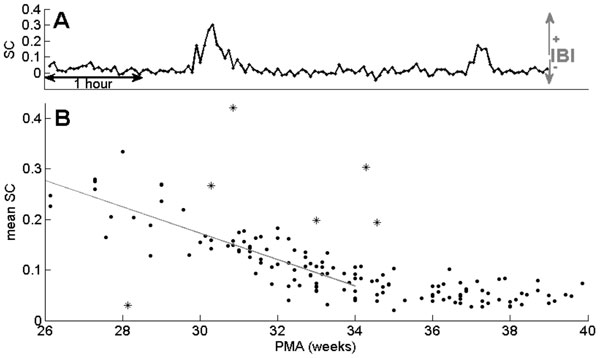
**A **Suppression curve example, containing 2 periods of 20-30 minutes of discontinuous pattern, **B **Evolution of the mean of the suppression curve in function of the age, · represents a patient with normal EEG, * patient with abnormal EEG

Automated analysis of premature electroencephalogram (EEG) for diagnosis is a crucial step to reduce the workload of neurologists. The grade of discontinuity gives important information about the maturation [1]. For normal maturation, the discontinuous pattern gradually evolves into a more continuous pattern. This means, interburst intervals (IBI), periods of low activity, become shorter. We have defined the suppression curve (SC), which is a "measure of discontinuity" [2] (Figure 1A). All data for this study were recorded at the Neonatal Intensive Care Unit, University Hospital Gasthuisberg, Leuven, Belgium. The dataset consisted of 170 EEG recordings (8 channels, 250 Hz) of 93 preterm infants with a postmenstrual age (PMA) of 24-40 weeks. Some maturational features are extracted from the discontinuous periods, like the IBI length and the synchrony index. However, the SC on itself gives also relevant information about the maturation. Taking the mean of every SC, we can find a correlation with the age till 34 weeks PMA (Figure 1B). Few outliers (abnormal EEG) are excluded. After that age, the patient is called late preterm or even term, and the EEG pattern is in normal condition mostly continuous (low values of the SC).

In conclusion, this research adds another valuable feature for the automated analysis of premature background EEG, which would improve the overall assessment in the NICU for EEG diagnosis
